# *Mycoplasma bovis*-associated verminous pneumonia in alpine chamois (*Rupicapra rupicapra*)

**DOI:** 10.3389/fvets.2024.1403682

**Published:** 2024-09-23

**Authors:** Michela Bullone, Sara Divari, Alessandra Sereno, Bruno Bassano, Daniela Gelmetti, Lucia Rita Gibelli, Paola Pregel, Enrico Bollo, Frine Eleonora Scaglione

**Affiliations:** ^1^Department of Veterinary Sciences, University of Turin, Turin, Italy; ^2^Gran Paradiso National Park, Alpine Wildlife Research Centre and Surveillance Service, Turin, Italy; ^3^Istituto Zooprofilattico Sperimentale della Lombardia e dell’Emilia-Romagna “Bruno Ubertini”, Brescia, Italy

**Keywords:** chamois, *Rupicapra rupicapra*, lung, *Mycoplasma bovis*, parasites, pneumonia

## Abstract

Pneumonia is a common disease affecting Alpine chamois. However, little is known concerning the etiological agents involved. We investigated whether *Mycoplasma* spp. infection occurs in Alpine chamois and describe the microscopic lesions associated with *Mycoplasma*-associated bronchopneumonia in this species. Lung tissues obtained from 45 chamois with gross evidence of pneumonia were analysed. The histological lesions and the presence of lungworms within the lungs were evaluated blindly. The presence of *Mycoplasma* spp. was assessed by immunohistochemistry (*Mycoplasma bovis* and *Mycoplasma mycoides* subsp*. mycoides*) and by end-point PCR. *M. bovis* was detected by immunohistochemistry and confirmed by PCR and sequencing in 6/45 (13%) cases, while all lungs were negative for *M. mycoides* subsp*. mycoides*. A significant association was found between the detection of *M. bovis* and the presence of severe lungworms infection in the examined lungs. We report for the first time *M. bovis* as a bacteria associated with verminous pneumonia in chamois.

## 1 Introduction

The chamois *Rupicapra* spp. is the most abundant mountain ungulate of Europe and the Near East, where it occurs as two species, the northern chamois *Rupicapra rupicapra* and the southern chamois *R. pyrenaica* (1). Although the chamois as a genus is not at risk, some populations have recently shown a decreasing trend, and some subspecies are threatened (2).

In the European Alps, infectious keratoconjunctivitis caused by *Mycoplasma conjunctivae,* a highly contagious ocular infection which is common in domestic sheep and goats, is often observed in Alpine chamois (*Rupicapra r. rupicapra*) (3). Moreover, several pathogens can affect these animals such bacteria (e.g., *Clamidiaceae),* viruses (e.g., Pestivirus) (4) or parasites (e.g., *Eimeria* spp.).

Also respiratory diseases are frequently detected in Alpine chamois (*Rupicapra rupicapra*) (5). Different pathogens have been identified in previous studies within the lungs of chamois living in Europe and causing pneumonia in this species (1, 2, 5–16).

Because interactions between livestock and chamois occur on Alpine pastures, transmission of infectious diseases is considered possible (4, 17). In the last decades, acute die-off of pneumonia in this species have been found due to bacterial bronchopneumonia caused by *Pasteurellaceae (Mannheimia glucosida, Bibersteinia trehalose)* in Austria (12). Moreover, *Nocardia otitidiscaviarum* have been diagnosed from the lung of an Alpine chamois with suppurative bronchopneumonia (8) and a *Respirovirus* genus, family *Paramamixoviridae*, has been identified in an Alpine chamois showing interstitial pneumonia associated with catarrhal bronchopneumonia (18).

Our research group recently became aware of a series of deaths due to *Mycoplasma bovis* infection in Alpine chamois in the Savoie region of south-eastern France (personal communication, Aguillar X.F.). The present work was therefore undertaken, retrospectively, to specifically investigate the presence and potential pathogenicity of *Mycoplasma* spp. in the lungs of Alpine chamois found dead in the neighbouring Italian Piedmont and Aosta Valley geographical area.

To the Authors’ best knowledge, *Mycoplasma* spp. infection has never been reported in wild chamois.

## 2 Methods

A retrospective observational study was performed using histological samples already available in the archives of the Pathology Service of the Department of Veterinary Sciences, University of Turin. The mentioned Pathology service is a reference point for monitoring local wild animal diseases and works in close collaboration with the national Veterinary Health Institutions, namely the network of Istituti Zooprofilattici Sperimentali. Within this framework, whole carcasses or isolated organ specimens from wild animals found dead are referred to the Pathology service from local veterinary observatories present in the Piedmont and Aosta Valley area (North-West Italy), for diagnostic purposes.

Lung tissues from chamois found dead in the Piedmont and Aosta Valley Mountain area from 1993 to 2010 were studied. In details, chamois retrieved from the following geographical areas were studied: Ossola Valley, Alpe Veglia e Alpe Devero Natural Park, veterinary observatories of the provinces of Biella, Cuneo and Turin, as well as Gran Paradiso National Park.

Lung samples were retrieved from the tissue archives of the previously mentioned Pathology service, after searching for “chamois” or “Rupicapra” as animal species, and “lung” as sampled organ in the database from the selected areas. For each animal, the necropsy report was consulted. Cases with complete necropsy report and ≥ 1 lung samples from an area showing gross lesions were selected.

The studied lung specimens had been harvested by experienced pathologist employed at the Pathology service (when entire cadavers were sent over), or by trained staff working in the field. In the latter case, a gross pathological examination preceded sample withdrawal; samples were then kept refrigerated and then formalin fixed as soon as possible and sent to the Pathology Service.

As the approximate time between the death of the animal and tissue fixation could not be determined, histologic slides were first scored for tissue quality using HE staining.

The experimental procedures described in the present paper are in accordance with relevant national regulation in terms of Ethics and Informed consent and have been judged as being free from the need of approval from the local Animal Welfare authority.

The presence of *M. bovis* and *M. mycoides* subsp*. mycoides* in the lung parenchyma was evaluated by immunohistochemistry (IHC) and by PCR and sequencing. Lastly, a thorough histological scoring of the lesions was performed on HE stained slides.

### 2.1 Immunohistochemistry

Immunohistochemical staining was performed on 3 μm formalin fixed (36–48 h) paraffin embedded serial sections after blocking endogenous peroxidase with 3% H_2_O_2_ for 10 min at room temperature. The tissues were then incubated overnight at 4°C with *M. bovis* or *M. mycoides* rabbit polyclonal antibodies (both produced by the IZSLER laboratories, Brescia, Italy), diluted 1:1500 and 1:8000, respectively. In accordance with manufacturer’s instructions, Novolink™ Polymer Detection Systems (Leica Biosystems) and NovaRED Peroxidase (HRP) Substrate Kit (Vector Laboratories) were applied. The sections were counterstained in Mayer’s Hematoxylin. Two negative controls were used: a bovine lung sample previously testing negative for *Mycoplasma* spp. and two bovine lung samples previously tested as positive to *M. bovis*/*M. mycoides* that were processed in absence of the primary antibodies. The same tissues yielded similar results when analysed by PCR for *Mycoplasma* spp. The two bovine samples with proven *M. bovis/M. mycoides* infection were used as positive controls. All control samples were provided by IZSLER laboratories.

### 2.2 PCR and sequencing

Given the potential serological cross-reactions between several *Mycoplasma* species, joined to the fact that *M. bovis* is not considered an infectious agent of *Caprinae*, most likely due to species specific adhesions like the proteins Vpma and P30, we performed further validation steps to validate our results (19).

First, as most reports that exist on *M. bovis* in *Caprinae* were then considered misidentifications with the closely related (both serologically and genetically) *M. agalactiae*, we repeated IHC on slides from ovine mastitis caused by *M. agalactiae* (clinical case confirmed by PCR diagnosis – we have tested 6 slides from 2 different cases, 3 slides each), and indeed the antibody we used for detecting *M. bovis* cross-reacted with *M. agalactiae*, yielding positive results and making it impossible to differentiate the two species only using IHC. Unfortunately, the paraffin blocks of these *M. agalactiae* cases were no longer available for further molecular analysis at this point (only archived slides, paraffin blocks no longer available).

We thus attempted a DNA extraction and species identification from formalin fixed and paraffin-embedded lung samples previously diagnosed with Mycoplasma infections in our study. Genomic DNA was obtained using E.Z.N.A.^®^ Tissue DNA Kit (Omega Bio-tek, Norcross, GA, US) from two or three sections of paraffin-embedded lung samples of about 20 μm thickness. The blade of the microtome was changed for each sample, in order to avoid cross-contamination and sections were previously treated with 1 mL of xylene followed to 1 mL of ethanol (96–100%) to extract residual xylene from the sample and then incubated at 37°C to remove all residual ethanol.

PCR was performed using primers genus-specific described by Choppa in 1998^3^ (forward: 5′ GGG AGC AAA CAG GAT TAG ATA CCC T 3′ and reverse: 5’ TGC ACC ATC TGT CAC TCT GTT AAC CTC 3′) and a master mix (HotStarTaq, Qiagen, Hilden, Germany). These primers amplify a 280 bp region in the genomic code of all species of mycoplasma. As 600 bp is considered the maximum size of DNA fragments amplifiable from formalin fixed and paraffin embedded samples, we choose to start using this approach.

We initially tested 4 samples from bovine clinical cases positive for *M. bovis* (positive controls, cases 1–4 in Supplementary Figure S1; Supplementary Table S2) and 2 samples from swine cases positive for *M. hyorhinis* (cases 5–6 in Supplementary Figure S1; Supplementary Table S2). These samples were sent to our Institute from the Pathology Service of the University of Montreal, with which we have a long date collaboration. PCR products were visualized by electrophoresis (Supplementary Figure S1), purified through MinElute PCR purification kit (Qiagen) and sequenced in both directions using Sanger method by a commercial sequencing provider (BMR Genomics, Padova, Italy).

The raw sequences were edited using Geneious Prime version 2021.2.2 and compared to sequences deposited in NCBI using BLAST.^1^

Lastly, we did PCR and sequenced the formalin fixed and paraffin embedded samples of chamois lung used for this work (initially only on all IHC positives [cases 1–6 in Supplementary Table S3], two IHC positives that were excluded from the study because of bad quality of the slides [cases 7–8 in Supplementary Table S3], and 2 IHC negative [cases 9–10 in Supplementary Table S3]; subsequently on all cases studied [Supplementary Table S4]).

### 2.3 Histology and scoring system

Histopathological lesions assessment was performed on 4 μm-thick formalin fixed paraffin embedded sections stained with hematoxylin and eosin (HE). Tissue quality was verified by visual assessment of stained slides.

Lesions observed in the pulmonary parenchyma, airways, vessels, and pleura were assessed on HE-stained samples by an experienced pathologist blinded to the animal ID. All lungs were assessed for lesion distribution (parenchyma, airways, pleura, or diffuse), for the presence of parasites (0: absent; 1: rare [<20 per slide]; 2: massive [≥20 per slide]), and for the type of inflammatory infiltrate (acute: mostly neutrophilic; chronic: mostly mononuclear cells; mixed: both neutrophils and mononuclear cells; eosinophilic: with a significantly increased eosinophilic component) or necrosis. Specifically, the lung parenchyma was evaluated for the presence, degree, and type (neutrophilic, eosinophilic, mononuclear cell) of interstitial and alveolar inflammation, interstitial thickening, parenchymal oedema, hemorrhage, hemosiderosis, emphysema, peribronchial metaplasia, and smooth muscle metaplasia. The presence of bronchial activated lymphoid tissue (BALT) was also verified. To this aim, we considered the presence of focal aggregates of lymphocytes organized into a follicle-like structure in the peribronchial region as activated BALT. Concerning the airways, alterations of the respiratory epithelium (desquamation/hyperplasia/metaplasia) and the presence, degree, and type of inflammation in the inner (lumen and epithelium) and outer (submucosa and adventitia) portion of the airways were assessed. Also, the presence of haemorrhages into the bronchial lumen, mucus gland hyperplasia/hypertrophy, and smooth muscle hyperplasia/hypertrophy were evaluated. Finally, the presence, degree, and type of inflammation of the vessels and of the pleura were analysed, together with the presence of hyperaemia and congestion of the tissue and with pleural thickening.

### 2.4 Statistical analysis

Statistical analyses were performed using Prism GraphPad Inc. v.6 on raw data. Chi-square tests were used to assess the presence of significant associations among the positivity to *M. bovis* and histological lesions or parasitic infection. Alpha was set at 0.05. Data are available from the corresponding author upon request.

## 3 Results

### 3.1 Lung tissue quality

The lung samples obtained from 5/50 chamois (10%) were of bad quality due to extensive postmortem changes and were excluded from the analysis. The remaining samples from 45 chamois were of good quality (with only mild to moderate postmortem changes), and were studied.

### 3.2 Immunohistochemistry

**Figure 1:**
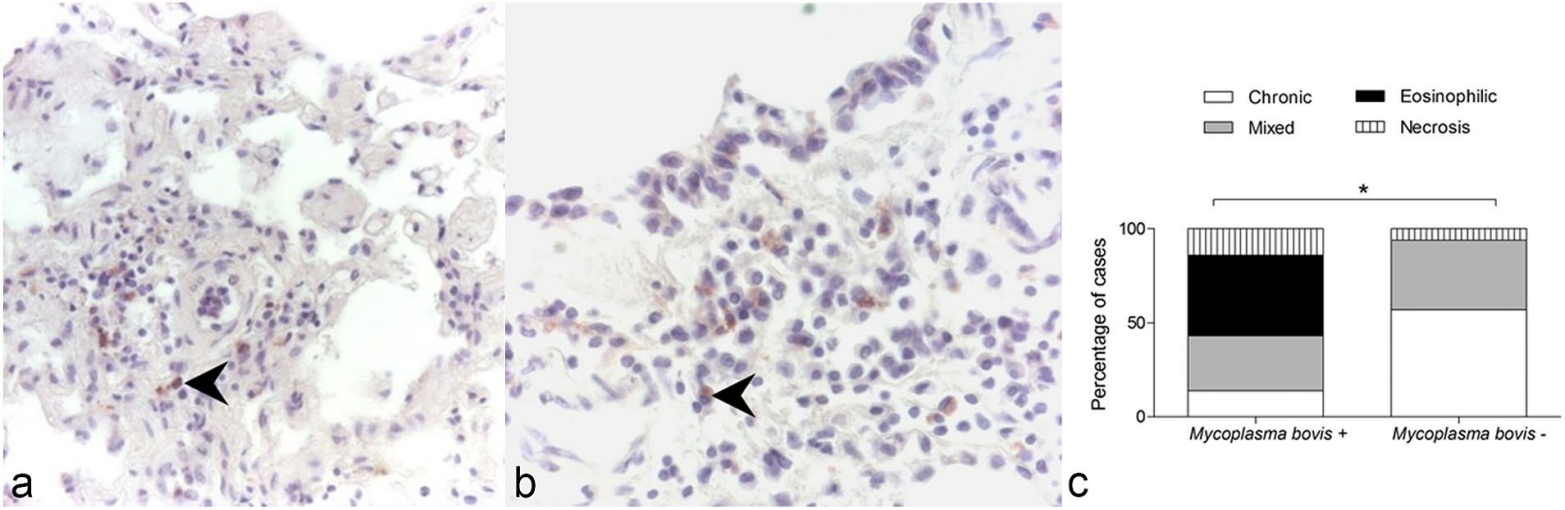
Immunohistochemistry for *Mycoplasma bovis*, lung, wild chamois. **(A,B)** Immunolabeling is present in mononuclear cells (arrows). **(C)** Association between the presence of *M. bovis* and the type of inflammatory infiltrate observed in the examined lung samples of wild chamois. The chi-square test revealed a significant association between these two variables. *p = 0.001.

At IHC, all samples tested negative for *M. mycoides* subsp*. mycoides*, while 6/45 animals (13%) resulted positive (Figures 1A,B) for the presence of *M. bovis*. Three of these samples were received in 1997 from the Ossola Valley region, 2 in 1999, and 1 in 2002 from the Turin area.

### 3.3 PCR and sequencing

PCR products sequencing correctly identified all 6 samples tested obtained from confirmed bovine and swine cases (Supplementary Table S2). Of note, although *M. bovis* was the known infectious agent and the microorganism with the highest degree of sequence identity, *M. agalactiae* as well had a high degree of identity. The strain identified by sequencing (NADC59) has been recently reported as a cause of lung pneumonia in cattle and bison in Canada (20).

Sequencing identified *M. bovis* as the microorganism observed in our chamois samples with the highest degree of sequence identity (comparable to the one obtained in our previous control run with confirmed bovine *M. bovis* samples, Supplementary Table S3). Specifically, there is a 4 bp difference between the two species which explain the lower % identity of *M. agalactiae* compared to *M. bovis* in our samples. Also, the strain of *M. bovis* identified is the same previously reported to cause pneumonia in cattle (20). We can also see here from PCR and sequencing results that the 2 IHC negative samples tested resulted in PCR positive and sequencing identified *M. bovis*, which is in line with the fact that PCR and sequencing are more sensitive techniques for pathogen identification compared to IHC. This was also reported by Radaelli in 2008 (13).

We thus performed DNA extraction, PCR amplification and sequencing for all samples included in our work (*n* = 45). Results are made available as Supplementary Table S4. Briefly, DNA of *M. bovis* was detected in 38/45 (84%) samples, with a sequence identity of 100% in 34/38 (89%) samples, >99% in 3/38 (8%) samples and 95.39% in 1/38 (3%) sample. In detail all IHC positive samples were positive also at PCR (100%), while *M. bovis* DNA was isolated from 32 out of 39 (82%) IHC negative samples. The 7 samples in which *M. bovis* DNA cannot be isolated yielded: no result due to low quality of sequencing in 5 cases, 1 sequence recognized as uncultured bacterium clone 1,103,200,819,504 16S ribosomal RNA gene, and 1 sequence confirmed as *M. arginini* (identity 98.95%).

### 3.4 Lung histology

Information concerning sex and age were not available for most animals.

**Figure 2:**
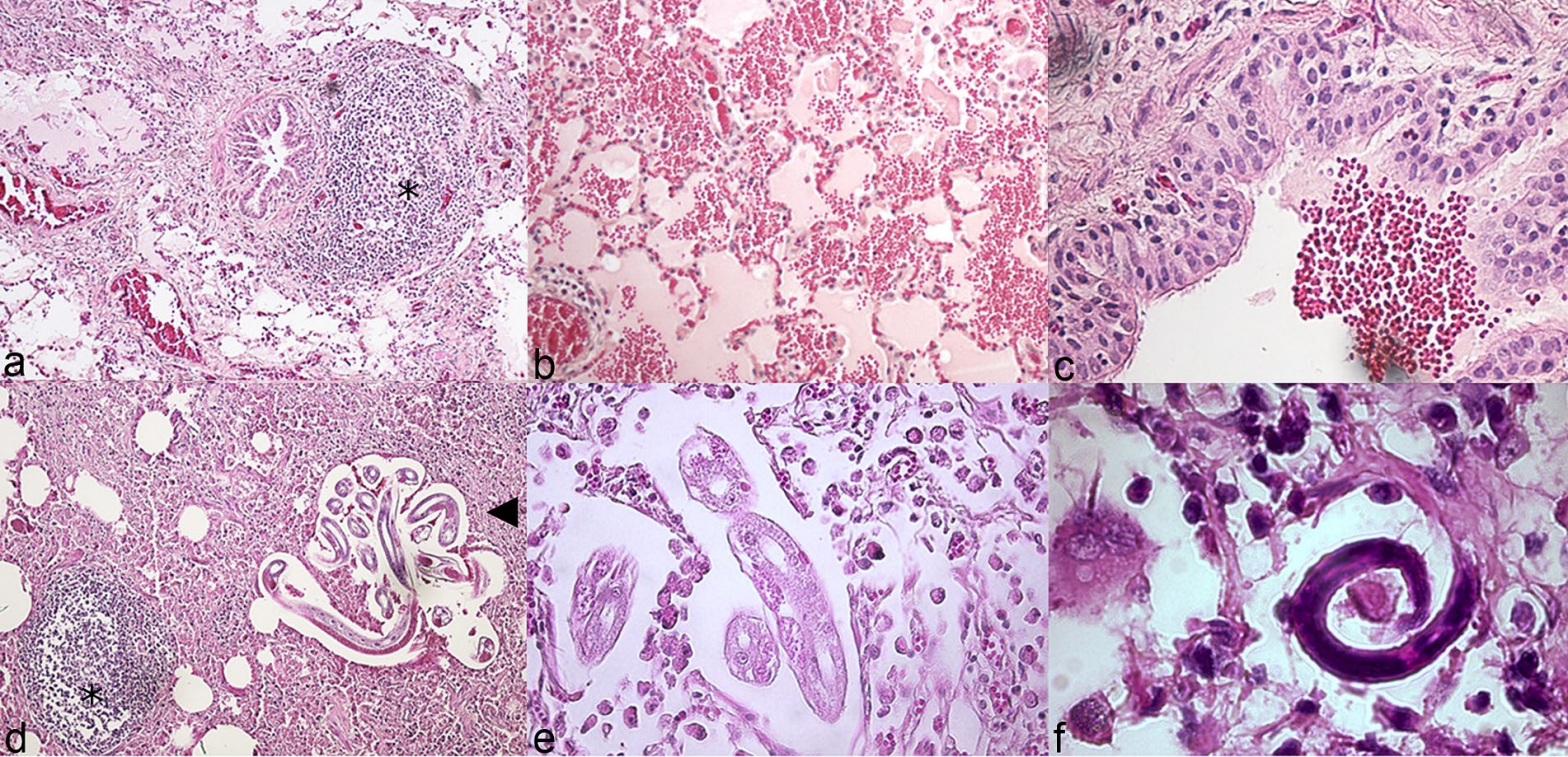
Histopathologic features of pneumonia, lung, wild chamois. Hematoxylin and eosin (HE). **(A)** Marked hyperplasia of peribronchial/bronchiolar activated lymphoid tissue (asterisk: BALT). **(B)** Alveolar hemorrhage. **(C)** Intrabronchial hemorrhage. **(D)** Parasites at different larval stages (arrowhead) and activated BALT (asterisk). **(E,F)** Details of lung parasites and associated eosinophilic inflammation.

Microscopic lesions associated with pneumonia in chamois were like those observed in other species (Supplementary Table S1). Marked hyperplasia of peribronchial/bronchiolar activated lymphoid tissue (BALT) was observed, causing in some cases partial obstruction of the airway lumen and collapse of the adjacent pulmonary parenchyma (referred to as lymphocytic “cuffing” pneumonia, Figure 2A). Peribronchial metaplasia was evident around most airways. Airway and alveolar accumulation of red blood cells was commonly observed, in association with hemosiderophages (Figures 2B,C). Lung parasites were often observed (Figures 2D–F).

The lungs testing positive for *M. bovis* at IHC presented exudative bronchopneumonia (involving most of the lung parenchyma and bronchial tree) with extensive necrotic foci surrounded by mononuclear and phagocytic inflammatory cells. Within the lung parenchyma, both acute and chronic inflammatory processes were identified, indicative of an active infection. The presence of eosinophilic inflammation was significantly associated with the identification of *M. bovis* at IHC in the lungs of alpine chamois (χ^2^ = 73.21, df 3; *p* = 0.001 after Bonferroni correction for multiple comparison, Figure 1C).

As *M. bovis* infection is not usually associated with eosinophilia, we investigated the possible causes of this finding. A significant association was found between the presence of *M. bovis* within the lung tissue and the presence of severe lung parasite infestation in these animals (χ^2^ = 7.73, df 2; *p* = 0.02). In detail, 4/6 lungs testing positive for *M. bovis* at IHC had microscopic evidence of high parasite burden at the lesion sites (coinfections), while this was observed in only 6/39 (16%) of the lungs where *M. bovis* was not detected by IHC. This could also explain the presence of extensive pulmonary hemorrhage, a finding not typically associated with *M. bovis* pneumonia (21, 22). Both adult worms and larval stages L3 and L4 were observed within the lung parenchyma of affected animals, belonging to the *Protostrongylidae* family (phylum Nematoda). Species identification was not possible due to incomplete imaging of the tail of the parasites in most images. Based on previous reports of parasitic infections in ruminants we expect them to belong to *Muelleris*, *Neostrongilus*, *Protostrongylus* and *Cystocaulus* spp.

## 4 Discussion

Pneumonia is common among wild chamois (7), and probably represents one of the main ways of natural selection in this species (10). Scarce data are available on etiology, although lungworms, bacteria (*Mannheimia*), and viruses likely play a role (1, 5, 8, 11, 15–17, 23). The knowledge of the agents involved in pneumonia complex in wild ruminants May be of value in the study of pathogens affecting grazing domestic animals and cattle sharing mountain pastures with this species. We investigated the presence and prevalence of *Mycoplasma* spp. in the lungs of chamois found dead in the Piedmont region of Italy with gross and microscopic evidence of pneumonia. This investigation was undertaken following the observation that the pathological lesions observed closely resembled those typically encountered in *Mycoplasma*-induced pneumonia in other species (21, 22). Our results provide the first evidence of the presence of *M. bovis* in the lungs of chamois affected by pneumonia, indicating *M. bovis* as a newly recognized pathogen of the alpine chamois. Whether this represents a new potential source of infection for domestic ruminants housed in the regions studied will have to be ascertained. *M. bovis* pulmonary infection in wild alpine chamois was associated with high lung parasite burden at the lesion site in our study. Whether localized high parasite burden occurs in consequence of the immunosuppressive effects of *M. bovis*, or *M. bovis* infection is facilitated because of the immunosuppression caused by severe parasitism remains to be established. In this regard, it is important to acknowledge that other stressing factors such as those related to climate, diet and concurrent infections can induce immunosuppression in wild animals. In the cases studied, which were randomly chosen, male and female chamois were equally represented and all except 3 were adult animals.

Monitoring circulating pathogens in wildlife is important because it allows the early detection and recognition of new etiological agents causing diseases, and also because it permits to control and prevent the transmission between wild and domestic animals and vice versa. This however must be coupled with a concurrent control of pathogens in domestic animals. Chamois are small ruminants, and they might be susceptible to the infection of agents commonly affecting the lungs of phylogenetically related species. *M. bovis* and *M. mycoides* subsp*. mycoides* May cause severe pneumonia in cattle and goats (21, 22, 24), respectively, and transmission to the Alpine chamois is considered possible based on host ecology and behavior (25). To the best of our knowledge, *Mycoplasma*-induced pneumonia has not been previously reported or investigated in Alpine chamois. *M. bovis*, a pathogen highly adapted to cattle where it can cause bronchopneumonia, mastitis, and arthritis (22), was detected by IHC in 13% of the lungs of chamois with gross and microscopic evidence of pneumonia in our study. Although this finding does not confirm *M. bovis* as the primary etiological agent causing pneumonia in these animals, nor the Alpine chamois as a potential reservoir of this microorganism, it shows that *M. bovis* can be harbored in the pulmonary parenchyma of wild chamois. Further work will have to verify the role of chamois in the epidemiology of *M. bovis* infection in wild and domestic animals in our geographical area.

Lung parasites commonly cause pneumonia in wild ruminants (5, 23). Infestations are often associated with secondary bacterial pneumonia (23), as severely parasitized animals are usually immunologically compromised. Also, *M. bovis* has been reported to be immunosuppressive (26), although this effect might be blunted or altered in chamois compared to cattle. This might explain why lung regions where *M. bovis* was detected had a higher parasite burden compared to the lung regions where *M. bovis* was not identified. The significant association found between the presence of *M. bovis* and pulmonary eosinophilic inflammation is likely due to the high parasitic burden found in these samples, and indicates an active response of the organism to it. Similarly, the high frequency of pulmonary necrotic lesions detected in samples positive for *M. bovis* strongly supports the pathogenicity of this microorganism in chamois and an active immune response of the organ to fight it. The pathological lesions observed in wild chamois with *M. bovis* infection are basically the same as those reported in naturally infected calves, where exudative bronchopneumonia and extensive foci of coagulative necrosis surrounded by inflammatory cells are observed (22, 27–29).

Limitation of the present study is the impossibility of testing all other pathogens inducing pneumonia in chamois on the selected samples due to the preservation method which not consent to perform bacteriological or virological exams.

## 5 Conclusion

In our study, *M. bovis* infection was associated with high pulmonary parasite burden in wild chamois, although causality of such coinfection remains to be determined. In conclusion, we report for the first time the presence of *M. bovis* in the lungs of chamois with histopathological traits of pneumonia. Whether this represents a risk for cattle and small ruminant herds commonly pastured within the same geographical areas remains unestablished.

## Data Availability

The datasets presented in this study can be found in online repositories. The names of the repository/repositories and accession number(s) can be found in the article/Supplementary material.

## Footnotes

^1^https://blast.ncbi.nlm.nih.gov/Blast.cgi
