# Supplementary material for: Mycoplasma bovis-associated verminous pneumonia in alpine chamois (Rupicapra rupicapra)
Source: Front Vet Sci. 2024 Sep 23;11:1403682. doi: 10.3389/fvets.2024.1403682 (PMC11456564; doi:10.3389/fvets.2024.1403682)
Supplement: Supplementary file 1 [file Table_1.DOCX]

ONLINE SUPPLEMENTARY MATERIAL

**MYCOPLASMA BOVIS-ASSOCIATED VERMINOUS PNEUMONIA IN ALPINE CHAMOIS (*Rupicapra rupicapra*)**

Michela Bullone^1^, Sara Divari^1^, Alessandra Sereno^1^, Bruno Bassano^2^, Daniela Gelmetti^3^, Lucia R. Gibelli^3^, Paola Pregel^1^, Enrico Bollo^1^, Frine E. Scaglione^1^.

Affiliations

^1^ Department of Veterinary Sciences, University of Turin, Largo Braccini 2, 10095 Grugliasco, Italy (MB, SD, AS, PP, EB, FES)

^2^ Gran Paradiso National Park, Alpine Wildlife Research Centre and Surveillance Service, Torino, Italy (BB)

^3^ Istituto Zooprofilattico Sperimentale della Lombardia e dell’Emilia-Romagna “Bruno Ubertini”, via Bianchi 9, 25124 Brescia, Italy (DG, LRG)

Corresponding author: Frine Eleonora Scaglione, Pathology Service, Department of Veterinary Sciences, University of Turin, Largo Braccini 2, 10095 Grugliasco (TO), Italy. Email: [frineeleonora.scaglione@unito.it](mailto:frineeleonora.scaglione@unito.it). Phone: +39 011 6709263.

**Supplementary figures and tables**

**
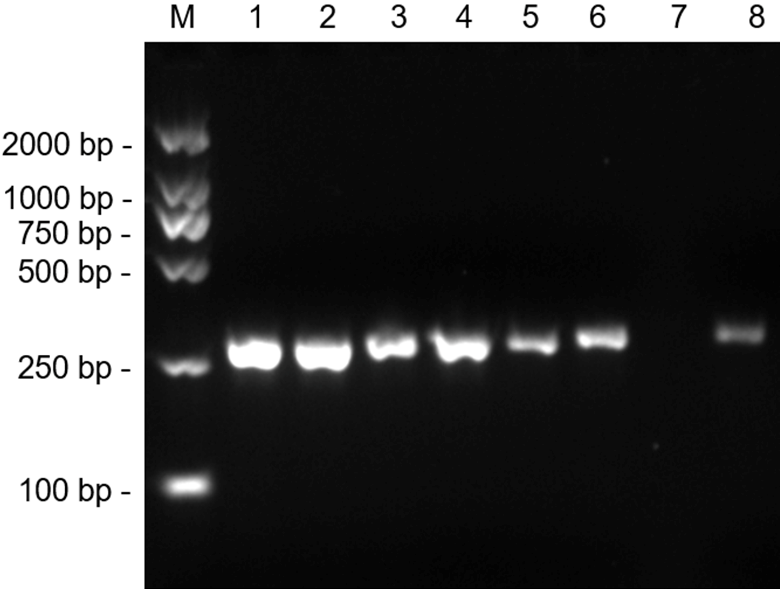
**

**Supplementary Figure S1.** Agarose gel electrophoresis of PCR amplified products generated from bovine clinical cases positive for *M. bovis* (lanes 1-4) and 2 samples from swine cases positive for *Mycoplasma hyorhinis* (lines 5-6). M: DNA size markers, GelPilot Mid-range ladder (Qiagen). Lane 7 was a negative (blank) control and lane 8 was *Mycoplasma* positive control (from *Mycoplasma* Detection Kit, Venor Gel Classic, Minerva Biolabs, Berlin, DE).

**Supplementary table S2.** Results obtained comparing PCR products nucleotide sequence of bovine and swine clinical cases positive for *M. bovis* (cases N. 1-4) and *M.* hyorhinis (cases N. 5-6), respectively, to sequence databases using BLAST.

| **Case N** | **bp** | **Sequence** | **Description** | **Max Score** | **Total Score** | **Query Cover %** | **E value** | **Ident %** | **GenBank Accession number** |
| --- | --- | --- | --- | --- | --- | --- | --- | --- | --- |
| 1 | 263 | GGGAGCAAACAGGATTAGATACCCTGGTAGTCCACGCCCTAAACGATGATCATTAGTTGATGGGGAACTCATCGACGCAGCTAACGCATTAAATGATCCGCCTGAGTAGTACGTTCGCAAGAATAAAACTTAAAGGAATTGACGGGGATCCGCACAAGCGGTGGAGCATGTGGTTTAATTTGATGTTACGCGTAGAACCTTACCCACTCTTGACATCTTCTGCAAAGCTATAGAGACATAGTGGAGGTTAACAGAGTGACAGT | *Mycoplasmopsis bovis* strain NADC59 chromosome, complete genome | 479 | 953 | 99 | 0 | 99,62 | CP042939.1 |
|  |  |  | *Mycoplasmopsis agalactiae* PG2 16S ribosomal RNA, partial sequence | 459 | 459 | 99 | 0 | 98,09 | NR_044667.2 |
| 2 | 263 | GGGAGCAAACAGGATTAGATACCCTGGTAGTCCACGCCCTAAACGATGATCATTAGTTGATGGGGAACTCATCGACGCAGCTAACGCATTAAATGATCCGCCTGAGTAGTACGTTCGCAAGAATAAAACTTAAAGGAATTGACGGGGATCCGCACAAGCGGTGGAGCATGTGGTTTAATTTGATGTTACGCGTAGAACCTTACCCACTCTTGACATCTTCTGCAAAGCTATRGAGACATAGTGGAGGTTAACAGAGTGACAGA | *Mycoplasmopsis bovis* strain NADC59 chromosome, complete genome | 477 | 955 | 100 | 0 | 99,24 | CP042939.1 |
|  |  |  | *Mycoplasmopsis* *agalactiae* PG2 16S ribosomal RNA, partial sequence | 462 | 462 | 100 | 0 | 98,1 | NR_044667.2 |
| 3 | 272 | TGGGAGCAAACAGGATTAGATACCCTGGTAGTCCACGCCCTAAACGATGATCATTAGTTGATGGGGAACTCATCGACGCAGCTAACGCATTAAATGATCCGCCTGAGTAGTACGTTCGCAAGAATAAAACTTAAAGGAATTGACGGGGATCCGCACAAGCGGTGGAGCATGTGGTTTAATTTGATGTTACGCGTAGAACCTTACCCACTCTTGACATCTTCTGCAAAGCTATAGAGACATAGTGGAGGTTAACAGAGTGACAGATGGTGCAA | *Mycoplasmopsis* *bovis* strain NADC59 chromosome, complete genome | 494 | 982 | 99 | 0 | 99,63 | CP042939.1 |
|  |  |  | *Mycoplasmopsis agalactiae* PG2 16S ribosomal RNA, partial sequence | 473 | 473 | 99 | 0 | 98,15 | NR_044667.2 |
| 4 | 272 | GGGAGCAAACAGGATTAGATACCCTGGTAGTCCACGCCSTAAACGATGATCATTAGTTGATGGGGAATTCATYGACGCAGCTAACGCATTAAATGATCCGCCTGAGTAGTACGTTCGCAAGAATAAAACTTAAAGGAATTGACGGGGATCCGCACAAGCGGTGGAGCATGTGGTTTAATTTGATGWTACGCGGTAGAACCTTACCCACTCTTGACATCTTTTGCAAAGCTATAGAGACATAGTGGAGGTTAACAGAGTGACAGATGGTGCAA | *Mycoplasmopsis bovis* strain NADC59 chromosome, complete genome | 466 | 927 | 99 | 0 | 97,42 | CP042939.1 |
|  |  |  | *Mycoplasmopsis agalactiae* PG2 16S ribosomal RNA, partial sequence | 451 | 451 | 99 | 0 | 96,31 | NR_044667.2 |
| 5 | 273 | TGGGAGCAAACAGGATTAGATACCCTGGTAGTCCACGCCGTAAACGATGATCATTAGTTGGTGGAATAATTTCACTAACGCAGCTAACGCGTTAAATGATCCGCCTGAGTAGTATGCTCGCAAGAGTGAAACTTAAAGGAATTGACGGGAACCCGCACAAGCGGTGGAGCATGTGGTTTAATTTGATGCTACGCGTAGAACCTTACCCACTCTTGACATCTTCTGCAAAGCTATAGAGATATAGTGGAGGTTAACAGAGTGACAGATGGTGCA | *Mycoplasmopsis hyorhinis* DBS 1050, complete genome | 492 | 492 | 99 | 0 | 99,26 | CP006849.1 |
|  |  |  | *Mycoplasmopsis agalactiae* PG2 16S ribosomal RNA, partial sequence | 375 | 375 | 99 | 0 | 91,54 | NR_044667.2 |
| 6 | 275 | TGGGAGCAAACAGGATTAGATACCCTGGTAGTCCACGCCGTAAACGATGATCATTAGTTGGTGGAATAATTTCACTAACGCAGCTAACGCGTTAAATGATCCGCCTGAGTAGTATGCTCGCAAGAGTGAAACTTAAAGGAATTGACGGGAACCCGCACAAGCGGTGGAGCATGTGGTTTAATTTGAATGCTACGCGTAGAACCTTACCCACTCTTGACATCTTCTGCAAAGCTATAGAGATATAGTGGAGGTTAACAGAGTGACAGATGGTGCAA | *Mesomycoplasma hyorhinis* strain USP83T 16S ribosomal RNA gene, partial sequence | 492 | 492 | 99 | 0 | 99,27 | GU227380.1 |
|  |  |  | *Mycoplasmopsis agalactiae* PG2 16S ribosomal RNA, partial sequence | 375 | 375 | 99 | 0 | 91,58 | NR_044667.2 |

**Supplementary Table S3.** Significant alignments obtained comparing PCR products sequence arising from *Mycoplasma* IHC positives sample (cases 1-6; samples 1a, 1b, and 1c are from different lung regions of the same animal), and *Mycoplasma* IHC negative sample (cases 7-8) to sequence databases using BLAST.

| **Case N** | **bp** | **Sequence** | **Description** | **Max Score** | **Total Score** | **Query Cover %** | **E value** | **% Ident** | **Accession** |
| --- | --- | --- | --- | --- | --- | --- | --- | --- | --- |
| 1a | 208 | TGGGAGCCAAACAGGATTAGATACCCTGGTAGTCCACGCCCTAAACGATGATCATTAGTTGATGGGGAACTCATCGACGCAGCTAACGCATTAAATGATCCGCCTGAGTAGTACGTTCGCAAGAATAAAACTTAAAGGAATTGACGGGGATCCGCACAAGCGGTGGAGCATGTGGTTTAATTTGATGTTACGCGTAGAACCTTACCCA | *Mycoplasmopsis bovis* strain NADC59 chromosome, complete genome | 375 | 751 | 99 | 0 | 99,52 | CP042939.1 |
|  |  |  | *Mycoplasmopsis agalactiae* isolate JF4428 genome assembly, chromosome: I | 364 | 729 | 99 | 0 | 98,55 | LT578418.1 |
| 1b | 263 | TGGGAGCAAACAGGATTAGATACCCTGGTAGTCCACGCCCTAAACGATGATCATTAGTTGATGGGGAACTCATCGACGCAGCTAACGCATTAAATGATCCGCCTGAGTAGTACGTTCGCAAGAATAAAACTTAAAGGAATTGACGGGGATCCGCACAAGCGGTGGAGCATGTGGTTTAATTTGATGTTACGCGTAGAACCTTACCCACTCTTGACATCTTCTGCAAAGCTATAGAGACATAGTGGAGGTTAACAGAGTGACAG | *Mycoplasmopsis bovis* strain NADC59 chromosome, complete genome | 479 | 953 | 99 | 0 | 99,62 | CP042939.1 |
| 1c | 270 | TGGGAGCAAACAGGATTAGATACCCTGGTAGTCCACGCCCTAAACGATGATCATTAGTTGATGGGGAACTCATCGACGCAGCTAACGCATTAAATGATCCGCCTGAGTAGTACGTTCGCAAGAATAAAACTTAAAGGAATTGACGGGGATCCGCACAAGCGGTGGAGCATGTGGTTTAATTTGATGTTACGCGTAGAACCTTACCCACTCTTGACATCTTCTGCAAAGCTATAGAGACATAGTGGAGGTTAACAGAGTGACAGTGGTGCA | *Mycoplasmopsis bovis* strain NADC59 chromosome, complete genome | 486 | 968 | 99 | 0 | 99,26 | CP042939.1 |
| 2 | 244 | TGTGGGAGCAAACAGGATTAGATACCGTGGTAGTCCACGCCCTAAACGATGATCATTAGTTGATGGGGAACTCATCGTACGCAGCTAACGCATTAAATGATCCGCCTGAGTAGTACGTTCGCAAGAATAAAACTTAAAGGAATTGACGGGGATCCGCACAAGCGGTGGAGCATGTGGTTTAATTTGATGTTACGCGTAGAACCTTACCCACTCTTGACATCTTCTGCAAAGCTATAGAGACATA | *Mycoplasmopsis bovis* strain NADC59 chromosome, complete genome | 433 | 860 | 98 | 0 | 99,17 | CP042939.1 |
|  |  |  | *Mycoplasmopsis agalactiae* PG2 chromosome, complete sequence | 416 | 833 | 98 | 0 | 97,93 | CU179680.1 |
| 3 | 263 | TGGGAGCAAACAGGATTAGATACCCTGGTAGTCCACGCCCTAAACGATGATCATTAGTTGATGGGGAACTCATCGACGCAGCTAACGCATTAAATGATCCGCCTGAGTAGTACGTTCGCAAGAATAAAACTTAAAGGAATTGACGGGGATCCGCACAAGCGGTGGAGCATGTGGTTTAATTTGATGTTACGCGTAGAACCTTACCCACTCTTGACATCTTCTGCAAAGCTATAGAGACATAGTGGAGGTTAACAGAGTGACAG | *Mycoplasmopsis bovis* strain NADC59 chromosome, complete genome | 479 | 953 | 99 | 0 | 99,62 | CP042939.1 |
|  |  |  | *Mycoplasmopsis agalactiae* strain 4136/96 no 2 16S ribosomal RNA gene, partial sequence | 464 | 464 | 99 | 0 | 98,47 | AF332750.1 |
|  |  |  | *Mycoplasmopsis agalactiae* strain 4136/96 no 2 16S ribosomal RNA gene, partial sequence | 464 | 464 | 99 | 0 | 98,47 | AF332750.1 |
| 4 | 262 | TGGGAGCAAACAGGATTAGATACCCTGGTAGTCCACGCCCTAAACGATGATCATTAGTTGATGGGGAACTCATCGACGCAGCTAACGCATTAAATGATCCGCCTGAGTAGTACGTTCGCAAGAATAAAACTTAAAGGAATTGACGGGGATCCGCACAAGCGGTGGAGCATGTGGTTTAATTTGATGTTACGCGTAGAACCTTACCCACTCTTGACATCTTCTGCAAAGCTATAGAGACATAGTGGAGGTTAACAGAGTGACA | *Mycoplasmopsis bovis* strain NADC59 chromosome, complete genome | 477 | 949 | 99 | 0 | 99,62 | CP042939.1 |
|  |  |  | *Mycoplasmopsis agalactiae* isolate JF4428 genome assembly, chromosome: I | 460 | 921 | 99 | 0 | 98,47 | LT578418.1 |
| 5 | 272 | TGGGAGCAAACAGGATTAGATACCCTGGTAGTCCACGCCCTAAACGATGATCATTAGTTGATGGGGAACTCATCGACGCAGCTAACGCATTAAATGATCCGCCTGAGTAGTACGTTCGCAAGAATAAAACTTAAAGGAATTGACGGGGATCCGCACAAGCGGTGGAGCATGTGGTTTAATTTGATGTTACGCGTAGAACCTTACCCACTCTTGACATCTTCTGCAAAGCTATAGAGACATAGTGGAGGTTAACAGAGTGACAGATGGTGCAA | *Mycoplasmopsis bovis* strain NADC59 chromosome, complete genome | 494 | 982 | 99 | 0 | 99,63 | CP042939.1 |
|  |  |  | *Mycoplasmopsis agalactiae* strain 4136/96 no 2 16S ribosomal RNA gene, partial sequence | 479 | 479 | 99 | 0 | 98,52 | AF332750.1 |
| 6 | 272 | TGGGAGCAAACAGGATTAGATACCCTGGTAGTCCACGCCCTAAACGATGATCATTAGTTGATGGGGAACTCATCGACGCAGCTAACGCATTAAATGATCCGCCTGAGTAGTACGTTCGCAAGAATAAAACTTAAAGGAATTGACGGGGATCCGCACAAGCGGTGGAGCATGTGGTTTAATTTGATGTTACGCGTAGAACCTTACCCACTCTTGACATCTTCTGCAAAGCTATAGAGACATAGTGGAGGTTAACAGAGTGACAGATGGTGCAA | *Mycoplasmopsis bovis* strain NADC59 chromosome, complete genome | 494 | 982 | 99 | 0 | 99,63 | CP042939.1 |
|  |  |  | *Mycoplasmopsis agalactiae* strain 4136/96 no 2 16S ribosomal RNA gene, partial sequence | 479 | 479 | 99 | 0 | 98,52 | AF332750.1 |
|  |  |  | *Mycoplasmopsis agalactiae* strain 4136/96 no 2 16S ribosomal RNA gene, partial sequence | 472 | 472 | 99 | 0 | 98,15 | AF332750.1 |
| 7 | 256 | TGTGGGAGCAAACAGGATTAGATACCCTGGTAGTCCACGCCCTAAACGATGATCATTAGTTGATGGGGAACTCATCGACGCAGCTAACGCATTAAATGATCCGCCTGAGTAGTACGTTCGCAAGAATAAAACTTAAAGGAATTGACGGGGATCCGCACAAGCGGTGGAGCATGTGGTTTAATTTGATGTTACGCGTAGAACCTTACCCACTCTTGACATCTTCTGCAAAGCTATATAGACATATTGGAGGTTAACA | *Mycoplasmopsis bovis* strain NADC59 chromosome, complete genome | 457 | 908 | 98 | 0 | 99,21 | CP042939.1 |
|  |  |  | *Mycoplasmopsis agalactiae* isolate JF4428 genome assembly, chromosome: I | 440 | 881 | 98 | 0 | 98,02 | LT578418.1 |
| 8 | 272 | TGGGAGCAAACAGGATTAGATACCCTGGTAGTCCACGCCCTAAACGATGATCATTAGTTGATGGGGAACTCATCGACGCAGCTAACGCATTAAATGATCCGCCTGAGTAGTACGTTCGCAAGAATAAAACTTAAAGGAATTGACGGGGATCCGCACAAGCGGTGGAGCATGTGGTTTAATTTGATGTTACGCGTAGAACCTTACCCACTCTTGACATCTTCTGCAAAGCTATAGAGACATAGTGGAGGTTAACAGAGTGACAGATGGTGCAA | *Mycoplasmopsis bovis* strain NADC59 chromosome, complete genome | 494 | 982 | 99 | 0 | 99,63 | CP042939.1 |
|  |  |  | *Mycoplasmopsis agalactiae* isolate JF4428 genome assembly, chromosome: I | 477 | 955 | 99 | 0 | 98,52 | LT578418.1 |

**Supplementary Table S4.** Significant alignments obtained comparing PCR products sequence arising from all cases studied to sequence databases using BLAST.

| **Slide ID** | **bp** | **Sequence** | **Description** | **Max Score** | **Total Score** | **Query cover %** | **E Value** | **Ident %** | **GenBank Accession number** |
| --- | --- | --- | --- | --- | --- | --- | --- | --- | --- |
| 1547/02a | 218 | GGTAGTCCACGCCCTAAACGATGATCATTAGTTGATGGGGAACTCATCGACGCAGCTAACGCATTAAATGATCCGCCTGAGTAGTACGTTCGCAAGAATAAAACTTAAAGGAATTGACGGGGATCCGCACAAGCGGTGGAGCATGTGGTTTAATTTGATGTTACGCGTAGAACCTTACCCACTCTTGACATCTTCTGCAAAGCTATAGAGACATAGTG | Mycoplasmopsis bovis strain NADC59 chromosome, complete genome | 403 | 801 | 100 | 0 | 100.00 | CP042939.1 |
| 1273/97 | 218 | GGTAGTCCACGCCCTAAACGATGATCATTAGTTGATGGGGAACTCATCGACGCAGCTAACGCATTAAATGATCCGCCTGAGTAGTACGTTCGCAAGAATAAAACTTAAAGGAATTGACGGGGATCCGCACAAGCGGTGGAGCATGTGGTTTAATTTGATGTTACGCGTAGAACCTTACCCACTCTTGACATCTTCTGCAAAGCTATAGAGACATAGTG | Mycoplasmopsis bovis strain NADC59 chromosome, complete genome | 403 | 801 | 100 | 0 | 100.00 | CP042939.1 |
| 1244/97 | 218 | GGTAGTCCACGCCCTAAACGATGATCATTAGTTGATGGGGAACTCATCGACGCAGCTAACGCATTAAATGATCCGCCTGAGTAGTACGTTCGCAAGAATAAAACTTAAAGGAATTGACGGGGATCCGCACAAGCGGTGGAGCATGTGGTTTAATTTGATGTTACGCGTAGAACCTTACCCACTCTTGACATCTTCTGCAAAGCTATAGAGACATAGTG | Mycoplasmopsis bovis strain NADC59 chromosome, complete genome | 403 | 801 | 100 | 0 | 100.00 | CP042939.1 |
| 1547/02b | 218 | GGTAGTCCACGCCCTAAACGATGATCATTAGTTGATGGGGAACTCATCGACGCAGCTAACGCATTAAATGATCCGCCTGAGTAGTACGTTCGCAAGAATAAAACTTAAAGGAATTGACGGGGATCCGCACAAGCGGTGGAGCATGTGGTTTAATTTGATGTTACGCGTAGAACCTTACCCACTCTTGACATCTTCTGCAAAGCTATAGAGACATAGTG | Mycoplasmopsis bovis strain NADC59 chromosome, complete genome | 403 | 801 | 100 | 0 | 100.00 | CP042939.1 |
| 172/99 | 218 | GGTAGTCCACGCCCTAAACGATGATCATTAGTTGATGGGGAACTCATCGACGCAGCTAACGCATTAAATGATCCGCCTGAGTAGTACGTTCGCAAGAATAAAACTTAAAGGAATTGACGGGGATCCGCACAAGCGGTGGAGCATGTGGTTTAATTTGATGTTACGCGTAGAACCTTACCCACTCTTGACATCTTCTGCAAAGCTATAGAGACATAGTG | Mycoplasmopsis bovis strain NADC59 chromosome, complete genome | 403 | 801 | 100 | 0 | 100.00 | CP042939.1 |
| 177/99 | 218 | GGTAGTCCACGCCCTAAACGATGATCATTAGTTGATGGGGAACTCATCGACGCAGCTAACGCATTAAATGATCCGCCTGAGTAGTACGTTCGCAAGAATAAAACTTAAAGGAATTGACGGGGATCCGCACAAGCGGTGGAGCATGTGGTTTAATTTGATGTTACGCGTAGAACCTTACCCACTCTTGACATCTTCTGCAAAGCTATAGAGACATAGTG | Mycoplasmopsis bovis strain NADC59 chromosome, complete genome | 403 | 801 | 100 | 0 | 100.00 | CP042939.1 |
| 1245/97 | 218 | GGTAGTCCACGCCCTAAACGATGATCATTAGTTGATGGGGAACTCATCGACGCAGCTAACGCATTAAATGATCCGCCTGAGTAGTACGTTCGCAAGAATAAAACTTAAAGGAATTGACGGGGATCCGCACAAGCGGTGGAGCATGTGGTTTAATTTGATGTTACGCGTAGAACCTTACCCACTCTTGACATCTTCTGCAAAGCTATAGAGACATAGTG | Mycoplasmopsis bovis strain NADC59 chromosome, complete genome | 403 | 801 | 100 | 0 | 100.00 | CP042939.1 |
| 1547/02c | 218 | GGTAGTCCACGCCCTAAACGATGATCATTAGTTGATGGGGAACTCATCGACGCAGCTAACGCATTAAATGATCCGCCTGAGTAGTACGTTCGCAAGAATAAAACTTAAAGGAATTGACGGGGATCCGCACAAGCGGTGGAGCATGTGGTTTAATTTGATGTTACGCGTAGAACCTTACCCACTCTTGACATCTTCTGCAAAGCTATAGAGACATAGTG | Mycoplasmopsis bovis strain NADC59 chromosome, complete genome | 403 | 801 | 100 | 0 | 100.00 | CP042939.1 |
| 148/10M | 218 | GGTAGTCCACGCCCTAAACGATGATCATTAGTTGATGGGGAACTCATCGACGCAGCTAACGCATTAAATGATCCGCCTGAGTAGTACGTTCGCAAGAATAAAACTTAAAGGAATTGACGGGGATCCGCACAAGCGGTGGAGCATGTGGTTTAATTTGATGTTACGCGTAGAACCTTACCCACTCTTGACATCTTCTGCAAAGCTATAGAGACATAGTG | Mycoplasmopsis bovis strain NADC59 chromosome, complete genome | 403 | 801 | 100 | 0 | 100.00 | CP042939.1 |
| 1734/09 | 218 | GGTAGTCCACGCCCTAAACGATGATCATTAGTTGATGGGGAACTCATCGACGCAGCTAACGCATTAAATGATCCGCCTGAGTAGTACGTTCGCAAGAATAAAACTTAAAGGAATTGACGGGGATCCGCACAAGCGGTGGAGCATGTGGTTTAATTTGATGTTACGCGTAGAACCTTACCCACTCTTGACATCTTCTGCAAAGCTATAGAGACATAGTG | Mycoplasmopsis bovis strain NADC59 chromosome, complete genome | 403 | 801 | 100 | 0 | 100.00 | CP042939.1 |
| 1245/97 | 218 | GGTAGTCCACGCCCTAAACGATGATCATTAGTTGATGGGGAACTCATCGACGCAGCTAACGCATTAAATGATCCGCCTGAGTAGTACGTTCGCAAGAATAAAACTTAAAGGAATTGACGGGGATCCGCACAAGCGGTGGAGCATGTGGTTTAATTTGATGTTACGCGTAGAACCTTACCCACTCTTGACATCTTCTGCAAAGCTATAGAGACATAGTG | Mycoplasmopsis bovis strain NADC59 chromosome, complete genome | 403 | 801 | 100 | 0 | 100.00 | CP042939.1 |
| 148/10M | 218 | GGTAGTCCACGCCCTAAACGATGATCATTAGTTGATGGGGAACTCATCGACGCAGCTAACGCATTAAATGATCCGCCTGAGTAGTACGTTCGCAAGAATAAAACTTAAAGGAATTGACGGGGATCCGCACAAGCGGTGGAGCATGTGGTTTAATTTGATGTTACGCGTAGAACCTTACCCACTCTTGACATCTTCTGCAAAGCTATAGAGACATAGTG | Mycoplasmopsis bovis strain NADC59 chromosome, complete genome | 403 | 801 | 100 | 0 | 100.00 | CP042939.1 |
| 1734/09 | 218 | GGTAGTCCACGCCCTAAACGATGATCATTAGTTGATGGGGAACTCATCGACGCAGCTAACGCATTAAATGATCCGCCTGAGTAGTACGTTCGCAAGAATAAAACTTAAAGGAATTGACGGGGATCCGCACAAGCGGTGGAGCATGTGGTTTAATTTGATGTTACGCGTAGAACCTTACCCACTCTTGACATCTTCTGCAAAGCTATAGAGACATAGTG | Mycoplasmopsis bovis strain NADC59 chromosome, complete genome | 403 | 801 | 100 | 0 | 100.00 | CP042939.1 |
| 1L 202/02 | 217 | GGTAGTCCACGCCCTAAACGATGATCATTAGTTGATGGGGAACTCATCGACGCAGCTAACGCATTAAATGATCCGCCTGAGTAGTACGTTCGCAAGAATAAAACTTAAAGGAATTGACGGGGATCCGCACAAGCGGTGGAGCATGTGGTTTAATTTGATGTTACGCGTAGAACCTTACCCACTCTTGACATCTTCTGCAAAGCTATAGAGACATAGT | Mycoplasmopsis bovis strain NADC59 chromosome, complete genome | 401 | 798 | 100 | 0 | 100.00 | CP042939.1 |
| 3L 309/02 | 217 | GGTAGTCCACGCCCTAAACGATGATCATTAGTTGATGGGGAACTCATCGACGCAGCTAACGCATTAAATGATCCGCCTGAGTAGTACGTTCGCAAGAATAAAACTTAAAGGAATTGACGGGGATCCGCACAAGCGGTGGAGCATGTGGTTTAATTTGATGTTACGCGTAGAACCTTACCCACTCTTGACATCTTCTGCAAAGCTATAGAGACATAGT | Mycoplasmopsis bovis strain NADC59 chromosome, complete genome | 401 | 798 | 100 | 0 | 100.00 | CP042939.1 |
| 4L 310/02 | 217 | GGTAGTCCACGCCCTAAACGATGATCATTAGTTGATGGGGAACTCATCGACGCAGCTAACGCATTAAATGATCCGCCTGAGTAGTACGTTCGCAAGAATAAAACTTAAAGGAATTGACGGGGATCCGCACAAGCGGTGGAGCATGTGGTTTAATTTGATGWTACGCGTAGAACCTTACCCACTCTTGACATCTTCTGCAAAGCTATAGAGACATAGT | Mycoplasmopsis bovis strain NADC59 chromosome, complete genome | 398 | 790 | 100 | 0 | 99.54 | CP042939.1 |
| 5L 311/02 |  |  | Low quality |  |  |  |  |  |  |
| 167/99 | 217 | GGTAGTCCACGCCGTAAACGATGATCATTAGTTGATGGAGAACTCATTGACGCAGCTAACGCATTAAATGATCCGCCTGAGTAGTATGCTCGCAAGAATAAAACTTAAAGGAATTGACGGGGATCCGCACAAGCGGTGGAGCATGTGGTTTAATTTGATGATACGCGGAGAACCTTACCCACTCTTGACATCCTTTGCAATGCTATAGAGACATAGT | Mycoplasmopsis bovis strain NADC59 chromosome, complete genome | 346 | 687 | 100 | 0 | 95.39 | CP042939.1 |
| 169/99 | 217 | GGTAGTCCACGCCCTAAACGATGATCATTAGTTGATGGGGAACTCATCGACGCAGCTAACGCATTAAATGATCCGCCTGAGTAGTACGTTCGCAAGAATAAAACTTAAAGGAATTGACGGGGATCCGCACAAGCGGTGGAGCATGTGGTTTAATTTGATGTTACGCGTAGAACCTTACCCACTCTTGACATCTTCTGCAAAGCTATAGAGACATAGT | Mycoplasmopsis bovis strain NADC59 chromosome, complete genome | 401 | 798 | 100 | 0 | 100.00 | CP042939.1 |
| 170/99 |  |  | Low quality |  |  |  |  |  |  |
| 171/99 | 218 | GGTAGTCCACGCCCTAAACGATGATCATTAGTTGATGGGGAACTCATCGACGCAGCTAACGCATTAAATGATCCGCCTGAGTAGTACGTTCGCAAGAATAAAACTTAAAGGAATTGACGGGGATCCGCACAAGCGGTGGAGCATGTGGTTTAATTTGATGTTACGCGTAGAACCTTACCCACTCTTGACATCTTCTGCAAAGCTATAGAGACATAGTG | Mycoplasmopsis bovis strain NADC59 chromosome, complete genome | 403 | 801 | 100 | 0 | 100.00 | CP042939.1 |
| 180/99 |  |  | Low quality |  |  |  |  |  |  |
| 181/99 | 191 | GGTAGTCCACGCCGTAAACGATGATCATTAGTCGGTGGAGAGTTCACTGACGCAGCTAACGCATTAAATGATCCGCCTGAGTAGTATGCTCGCAAGAGTGAAACTTAAAGGAATTGACGGGGACCCGCACAAGCGGTGGAGCATGTGGTTTAATTTGATGATACGCGTAGAACCTTACCCACTCTTGACAT | Mycoplasmopsis arginini strain MYCO17 16S ribosomal RNA gene, | 342 | 342 | 100 | 0 | 98.95 | MK789491.1 |
| 182/99 | 218 | GGTAGTCCACGCCCTAAACGATGATCATTAGTTGATGGGGAACTCATCGACGCAGCTAACGCATTAAATGATCCGCCTGAGTAGTACGTTCGCAAGAATAAAACTTAAAGGAATTGACGGGGATCCGCACAAGCGGTGGAGCATGTGGTTTAATTTGATGTTACGCGTAGAACCTTACCCACTCTTGACATCTTCTGCAAAGCTATAGAGACATAGTG | Mycoplasmopsis bovis strain NADC59 chromosome, complete genome | 403 | 801 | 100 | 0 | 100.00 | CP042939.1 |
| 185/99 | 218 | GGTAGTCCACGCCCTAAACGATGATCATTAGTTGATGGGGAACTCATCGACGCAGCTAACGCATTAAATGATCCGCCTGAGTAGTACGTTCGCAAGAATAAAACTTAAAGGAATTGACGGGGATCCGCACAAGCGGTGGAGCATGTGGTTTAATTTGATGTTACGCGTAGAACCTTACCCACTCTTGACATCTTCTGCAAAGCTATAGAGACATAGTG | Mycoplasmopsis bovis strain NADC59 chromosome, complete genome | 403 | 801 | 100 | 0 | 100.00 | CP042939.1 |
| 186/99 | 218 | GGTAGTCCACGCCcTAAACGATGATCATTAGTTGATGGGGAACTCATcGACGCAGCTAACGCATTAAATGATCCGCCTGAGTAGTAcGTTCGCAAGAATAAAACTTAAAGGAATTGACGGGGATCCGCACAAGCGGTGGAGCATGTGGTTTAATTTGATGATACGCGTAGAACCTTACCCACTCTTGACATCTTCTGCAAAGCTATAGAGACATAGTG | Mycoplasmopsis bovis strain NADC59 chromosome, complete genome | 398 | 790 | 100 | 0 | 99.54 | CP042939.1 |
| 190/99 | 218 | GGTAGTCCACGCCCTAAACGATGATCATTAGTTGATGGGGAACTCATCGACGCAGCTAACGCATTAAATGATCCGCCTGAGTAGTACGTTCGCAAGAATAAAACTTAAAGGAATTGACGGGGATCCGCACAAGCGGTGGAGCATGTGGTTTAATTTGATGTTACGCGTAGAACCTTACCCACTCTTGACATCTTCTGCAAAGCTATAGAGACATAGTG | Mycoplasmopsis bovis strain NADC59 chromosome, complete genome | 403 | 801 | 100 | 0 | 100.00 | CP042939.1 |
| 196/99 | 218 | GGTAGTCCACGCCCTAAACGATGATCATTAGTTGATGGGGAACTCATCGACGCAGCTAACGCATTAAATGATCCGCCTGAGTAGTACGTTCGCAAGAATAAAACTTAAAGGAATTGACGGGGATCCGCACAAGCGGTGGAGCATGTGGTTTAATTTGATGTTACGCGTAGAACCTTACCCACTCTTGACATCTTCTGCAAAGCTATAGAGACATAGTG | Mycoplasmopsis bovis strain NADC59 chromosome, complete genome | 403 | 801 | 100 | 0 | 100.00 | CP042939.1 |
| 198/99 | 218 | GGTAGTCCACGCCCTAAACGATGATCATTAGTTGATGGGGAACTCATCGACGCAGCTAACGCATTAAATGATCCGCCTGAGTAGTACGTTCGCAAGAATAAAACTTAAAGGAATTGACGGGGATCCGCACAAGCGGTGGAGCATGTGGTTTAATTTGATGTTACGCGTAGAACCTTACCCACTCTTGACATCTTCTGCAAAGCTATAGAGACATAGTG | Mycoplasmopsis bovis strain NADC59 chromosome, complete genome | 403 | 801 | 100 | 0 | 100.00 | CP042939.1 |
| 199/99 | 218 | GGTAGTCCACGCCCTAAACGATGATCATTAGTTGATGGGGAACTCATCGACGCAGCTAACGCATTAAATGATCCGCCTGAGTAGTACGTTCGCAAGAATAAAACTTAAAGGAATTGACGGGGATCCGCACAAGCGGTGGAGCATGTGGTTTAATTTGATGTTACGCGTAGAACCTTACCCACTCTTGACATCTTCTGCAAAGCTATAGAGACATAGTG | Mycoplasmopsis bovis strain NADC59 chromosome, complete genome | 403 | 801 | 100 | 0 | 100.00 | CP042939.1 |
| 201/99 | 218 | GGTAGTCCACGCCCTAAACGATGATCATTAGTTGATGGGGAACTCATCGACGCAGCTAACGCATTAAATGATCCGCCTGAGTAGTACGTTCGCAAGAATAAAACTTAAAGGAATTGACGGGGATCCGCACAAGCGGTGGAGCATGTGGTTTAATTTGATGTTACGCGTAGAACCTTACCCACTCTTGACATCTTCTGCAAAGCTATAGAGACATAGTG | Mycoplasmopsis bovis strain NADC59 chromosome, complete genome | 403 | 801 | 100 | 0 | 100.00 | CP042939.1 |
| 203/99 | 218 | GGTAGTCCACGCCCTAAACGATGATCATTAGTTGATGGGGAACTCATCGACGCAGCTAACGCATTAAATGATCCGCCTGAGTAGTACGTTCGCAAGAATAAAACTTAAAGGAATTGACGGGGATCCGCACAAGCGGTGGAGCATGTGGTTTAATTTGATGTTACGCGTAGAACCTTACCCACTCTTGACATCTTCTGCAAAGCTATAGAGACATAGTG | Mycoplasmopsis bovis strain NADC59 chromosome, complete genome | 403 | 801 | 100 | 0 | 100.00 | CP042939.1 |
| 204/99 | 216 | GGTAGTCCACGCCCTAAACGATGATCATTAGTTGATGGGGAACTCATCGACGCAGCTAACGCATTAAATGATCCGCCTGAGTAGTACGTTCGCAAGAATAAAACTTAAAGGAATTGACGGGGATCCGCACAAGCGGTGGAGCATGTGGTTTAATTTGATGTTACGCGTAGAACCTTACCCACTCTTGACATCTTCTGCAAAGCTATAGAGACATAG | Mycoplasmopsis bovis strain NADC59 chromosome, complete genome | 399 | 794 | 100 | 0 | 100.00 | CP042939.1 |
| 208/99 | 218 | GGTAGTCCACGCCCTAAACGATGATCATTAGTTGATGGGGAACTCATCGACGCAGCTAACGCATTAAATGATCCGCCTGAGTAGTACGTTCGCAAGAATAAAACTTAAAGGAATTGACGGGGATCCGCACAAGCGGTGGAGCATGTGGTTTAATTTGATGTTACGCGTAGAACCTTACCCACTCTTGACATCTTCTGCAAAGCTATAGAGACATAGTG | Mycoplasmopsis bovis strain NADC59 chromosome, complete genome | 403 | 801 | 100 | 0 | 100.00 | CP042939.1 |
| 550/99 | 217 | AGTAGTCCACGCCGTAAACGATGAGTACTAAGTGTCGGGCAACCGGTGCTGAAGTTAACACATTAAGTACTCCGCCTGAGTAGTACGGTCGCAAGGCTGAAACTCAAAGGAATTGACGGGCACCCGCACAAGCGGTGGAGCATGCTGTTTAATTCGAAACTACGCGAAGAACCTTACCTAGGTTTGACATCCCCGGCAAAGCCATGGAAACATAGTG | Uncultured bacterium clone 1103200819504 16S ribosomal RNA gen | 385 | 385 | 100 | 0 | 98.62 | EU842209.1 |
| 1855/99 |  |  | Low quality |  |  |  |  |  |  |
| 185/00 | 218 | GGTAGTCCACGCCCTAAACGATGATCATTAGTTGATGGGGAACTCATCGACGCAGCTAACGCATTAAATGATCCGCCTGAGTAGTACGTTCGCAAGAATAAAACTTAAAGGAATTGACGGGGATCCGCACAAGCGGTGGAGCATGTGGTTTAATTTGATGTTACGCGTAGAACCTTACCCACTCTTGACATCTTCTGCAAAGCTATAGAGACATAGTG | Mycoplasmopsis bovis strain NADC59 chromosome, complete genome | 403 | 801 | 100 | 0 | 100.00 | CP042939.1 |
| 1731/09 | 218 | GGTAGTCCACGCCCTAAACGATGATCATTAGTTGATGGGGAACTCATCGACGCAGCTAACGCATTAAATGATCCGCCTGAGTAGTACGTTCGCAAGAATAAAACTTAAAGGAATTGACGGGGATCCGCACAAGCGGTGGAGCATGTGGTTTAATTTGATGTTACGCGTAGAACCTTACCCACTCTTGACATCTTCTGCAAAGCTATAGAGACATAGTG | Mycoplasmopsis bovis strain NADC59 chromosome, complete genome | 403 | 801 | 100 | 0 | 100.00 | CP042939.1 |
| 1732/09 | 218 | GGTAGTCCACGCCCTAAACGATGATCATTAGTTGATGGGGAACTCATCGACGCAGCTAACGCATTAAATGATCCGCCTGAGTAGTACGTTCGCAAGAATAAAACTTAAAGGAATTGACGGGGATCCGCACAAGCGGTGGAGCATGTGGTTTAATTTGATGTTACGCGTAGAACCTTACCCACTCTTGACATCTTCTGCAAAGCTATAGAGACATAGTG | Mycoplasmopsis bovis strain NADC59 chromosome, complete genome | 403 | 801 | 100 | 0 | 100.00 | CP042939.1 |
| 1733/09 | 218 | GGTAGTCCACGCCCTAAACGATGATCATTAGTTGATGGGGAACTCATCGACGCAGCTAACGCATTAAATGATCCGCCTGAGTAGTACGTTCGCAAGAATAAAACTTAAAGGAATTGACGGGGATCCGCACAAGCGGTGGAGCATGTGGTTTAATTTGATGTTgCGCGTAGAACCTTACCCACTCTTGACATCTcCTGCAAAGCTATAGAGACATAGTG | Mycoplasmopsis bovis strain NADC59 chromosome, complete genome | 392 | 779 | 100 | 0 | 99.08 | CP042939.1 |
| 1735/09 | 218 | GGTAGTCCACGCCCTAAACGATGATCATTAGTTGATGGGGAACTCATCGACGCAGCTAACGCATTAAATGATCCGCCTGAGTAGTACGTTCGCAAGAATAAAACTTAAAGGAATTGACGGGGATCCGCACAAGCGGTGGAGCATGTGGTTTAATTTGATGTTACGCGTAGAACCTTACCCACTCTTGACATCTTCTGCAAAGCTATAGAGACATAGTG | Mycoplasmopsis bovis strain NADC59 chromosome, complete genome | 403 | 801 | 100 | 0 | 100.00 | CP042939.1 |
| 1736/09 | 218 | GGTAGTCCACGCCCTAAACGATGATCATTAGTTGATGGGGAACTCATCGACGCAGCTAACGCATTAAATGATCCGCCTGAGTAGTACGTTCGCAAGAATAAAACTTAAAGGAATTGACGGGGATCCGCACAAGCGGTGGAGCATGTGGTTTAATTTGATGTTACGCGTAGAACCTTACCCACTCTTGACATCTTCTGCAAAGCTATAGAGACATAGTG | Mycoplasmopsis bovis strain NADC59 chromosome, complete genome | 403 | 801 | 100 | 0 | 100.00 | CP042939.1 |
| 1737/09 |  |  | Low quality |  |  |  |  |  |  |
| 1738/09 | 218 | GGTAGTCCACGCCCTAAACGATGATCATTAGTTGATGGGGAACTCATCGACGCAGCTAACGCATTAAATGATCCGCCTGAGTAGTACGTTCGCAAGAATAAAACTTAAAGGAATTGACGGGGATCCGCACAAGCGGTGGAGCATGTGGTTTAATTTGATGTTACGCGTAGAACCTTACCCACTCTTGACATCTTCTGCAAAGCTATAGAGACATAGTG | Mycoplasmopsis bovis strain NADC59 chromosome, complete genome | 403 | 801 | 100 | 0 | 100.00 | CP042939.1 |
| 1739/09 | 218 | GGTAGTCCACGCCCTAAACGATGATCATTAGTTGATGGGGAACTCATCGACGCAGCTAACGCATTAAATGATCCGCCTGAGTAGTACGTTCGCAAGAATAAAACTTAAAGGAATTGACGGGGATCCGCACAAGCGGTGGAGCATGTGGTTTAATTTGATGTTACGCGTAGAACCTTACCCACTCTTGACATCTTCTGCAAAGCTATAGAGACATAGTG | Mycoplasmopsis bovis strain NADC59 chromosome, complete genome | 403 | 801 | 100 | 0 | 100.00 | CP042939.1 |
| 1740/09 | 218 | GGTAGTCCACGCCCTAAACGATGATCATTAGTTGATGGGGAACTCATCGACGCAGCTAACGCATTAAATGATCCGCCTGAGTAGTACGTTCGCAAGAATAAAACTTAAAGGAATTGACGGGGATCCGCACAAGCGGTGGAGCATGTGGTTTAATTTGATGTTACGCGTAGAACCTTACCCACTCTTGACATCTTCTGCAAAGCTATAGAGACATAGTG | Mycoplasmopsis bovis strain NADC59 chromosome, complete genome | 403 | 801 | 100 | 0 | 100.00 | CP042939.1 |
| 148-a/10 | 218 | GGTAGTCCACGCCCTAAACGATGATCATTAGTTGATGGGGAACTCATCGACGCAGCTAACGCATTAAATGATCCGCCTGAGTAGTACGTTCGCAAGAATAAAACTTAAAGGAATTGACGGGGATCCGCACAAGCGGTGGAGCATGTGGTTTAATTTGATGTTACGCGTAGAACCTTACCCACTCTTGACATCTTCTGCAAAGCTATAGAGACATAGTG | Mycoplasmopsis bovis strain NADC59 chromosome, complete genome | 403 | 801 | 100 | 0 | 100.00 | CP042939.1 |
